# Supervised Exercise Therapy Using Mobile Health Technology in Patients With Peripheral Arterial Disease: Pilot Randomized Controlled Trial

**DOI:** 10.2196/24214

**Published:** 2021-08-16

**Authors:** Katrin Paldán, Martin Steinmetz, Jan Simanovski, Christos Rammos, Greta Ullrich, Rolf Alexander Jánosi, Susanne Moebus, Tienush Rassaf, Julia Lortz

**Affiliations:** 1 Institute for Urban Public Health University Clinic of Essen University of Duisburg-Essen, Germany Essen Germany; 2 Personal Analytics Centre of Competence Department of Engineering Sciences University of Duisburg-Essen Essen Germany; 3 Department of Cardiology and Vascular Medicine West-German Heart and Vascular Center Essen University of Duisburg-Essen Essen Germany

**Keywords:** peripheral arterial disease, mHealth, supervised exercise therapy, motivation, artery, exercise, mobile applications, lifestyle, well-being

## Abstract

**Background:**

Mobile health interventions are intended to support complex health care needs in chronic diseases digitally, but they are mainly targeted at general health improvement and neglect disease-specific requirements. Therefore, we designed TrackPAD, a smartphone app to support supervised exercise training in patients with peripheral arterial disease.

**Objective:**

This pilot study aimed to evaluate changes in the 6-minute walking distance (meters) as a primary outcome measure. The secondary outcome measures included changes in physical activity and assessing the patients’ peripheral arterial disease–related quality of life.

**Methods:**

This was a pilot two-arm, single-blinded, randomized controlled trial. Patients with symptomatic PAD (Fontaine stage IIa/b) and access to smartphones were eligible. Eligible participants were randomly assigned to the study, with the control group stratified by the distance covered in the 6-minute walking test using the TENALEA software. Participants randomized to the intervention group received usual care and the mobile intervention (TrackPAD) for the follow-up period of 3 months, whereas participants randomized to the control group received routine care only. TrackPAD records the frequency and duration of training sessions and pain levels using manual user input. Clinical outcome data were collected at the baseline and after 3 months via validated tools (the 6-minute walk test and self-reported quality of life). The usability and quality of the app were determined using the Mobile Application Rating Scale user version.

**Results:**

The intervention group (n=19) increased their mean 6-minute walking distance (83 meters, SD 72.2), while the control group (n=20) decreased their mean distance after 3 months of follow-up (–38.8 meters, SD 53.7; *P*=.01). The peripheral arterial disease–related quality of life increased significantly in terms of “symptom perception” and “limitations in physical functioning.” Users’ feedback showed increased motivation and a changed attitude toward performing supervised exercise training.

**Conclusions:**

Besides the rating providing a valuable support tool for the user group, the mobile intervention TrackPAD was linked to a change in prognosis-relevant outcome measures combined with enhanced coping with the disease. The influence of mobile interventions on long-term prognosis must be evaluated in the future.

**Trial Registration:**

ClinicalTrials.gov NCT04947228; https://clinicaltrials.gov/ct2/show/NCT04947228

## Introduction

### Background

The circulatory disorders of peripheral arteries due to atherosclerotic lesions, also known as peripheral arterial diseases (PAD), are the third most frequent manifestations of cardiovascular diseases (CVD) after coronary and cerebrovascular arterial diseases [1].

A primary goal in CVD treatment is to slow down disease progression and avoid major adverse cardiac or limb events. Nonetheless, patients with PAD lag behind those with coronary artery disease (CAD) in terms of optimal treatment patterns [2-4]. Although the survival rates for CAD and PAD have improved worldwide, PAD still comes with a high individual burden regarding the quality of life (QoL) and associated disabilities [2].

The individual restrictions in the daily life of patients with PAD are more important than statistical facts regarding mortality and morbidity. Intermittent claudication causes a progressive reduction of the pain-free walking distance (PWD), and it is an expression of worsening PAD [4]. This decrease in physical capability results in declining mental health and reduces patients’ QoL [5].

Supervised exercise therapy (SET) is a cornerstone in the conservative management of intermittent claudication [4], and it extends the PWD. Even though SET is easy to practice and highly cost-effective, adherence to regular SET performance is relatively low [6,7]. The underuse of exercise can be partly explained by the lack of institutional resources [8] and both patients’ and physicians’ lack of interest in exercise [4,9].

Mobile health (mHealth) technologies increase incentives and provide digital support for patients with PAD on several treatment levels [10-12]. They potentially lead to higher exercise training adherence and widen the scope of patient-centered health care [13], but so far, studies show opposite results [11,14]. While patients with PAD highly desire specific support tools, and app stores are inundated with health and fitness apps, PAD-specific solutions are presently lacking [15].

### Objective

We developed a smartphone app named TrackPAD [16] to provide PAD-specific support for SET. This pilot study aims to evaluate the TrackPAD application as to its suitability and feasibility in outcome measures relevant to the prognosis of PAD by assessing the participant’s 6-minute walking distance (meters).

## Methods

### Study Aims, Research Questions, and Outcomes

The TrackPAD pilot study aimed to answer the following research questions:

1. Is it feasible to implement the app into everyday practice?
2. Is TrackPAD suitable for recording patients’ daily and weekly SET performance?
3. Does the TrackPAD improve the prognosis of PAD and related QoL?

The primary outcome was defined as the change in the 6-minute walking distance using a standardized protocol at baseline and after 3 months of follow-up [17]. The 6-minute walk test was performed under the supervision of a trained exercise technician. Participants were instructed to cover as much distance as possible, walking up and down a 50-meter hallway for up to 6 minutes. Participants were asked to push a measuring wheel during the entire 6 minutes of the test, but they could take breaks if necessary. They were also allowed to use an assistive device during both walking tests if they so desired. The technician stood in the middle of the course and supervised the walking test, but they did not encourage participants. The total distance walked in the test was read off of the measuring wheel. In patients with heart failure and reduced ejection fraction, a decreased 6-minute walking distance was associated with increased mortality, nonfatal cardiovascular events, and heart failure–related hospitalizations [18-20]. A decreased 6-minute walking distance was associated with a predictive value of mortality in patients with chronic obstructive pulmonary disease [21]. Among patients with PAD, the baseline 6-minute walking distance predicts rates for all-cause mortality, CVD mortality, and mobility loss [22,23]. Additionally, an incline of 20 meters was linked to a considerable improvement in total walking ability [24].

Aside from the 6-minute walking distance being objective and well-validated with respect to walking ability predicting mobility loss and mortality in PAD, it has an excellent test-retest reliability [25,26]. The 6-minute walking test offers several advantages over treadmill testing in PAD as it correlates more closely with physical activity levels and is not associated with the learning effect of performing repeated tests [27].

The secondary outcome measures were changes in physical activity and assessing the patient’s PAD-related QoL via PAD-QoL. The PAD-QoL questionnaire is a validated PAD-specific questionnaire [28] containing five factors: (1) social relationships and interactions, (2) self-concept and feelings, (3) symptoms and limitations in physical functioning, (4) fear and uncertainty, and (5) positive adaptation. In addition, individual factors regarding sexual function, intimate relationships, and job function will also be assessed. An evaluation of the use of the TrackPAD app was also performed for the intervention group using the user version of the Mobile Application Rating Scale questionnaire. It provides a 20-item measure including 4 objective quality subscales for engagement, functionality, aesthetics, and information quality [29].

### Study Design, Population, and the TrackPAD App

#### Study Design and Recruitment

This paper reports the results from the pilot study, including TrackPAD app usability tests for the target group (patients with PAD). In preparation for the pilot study, we conducted a recently published questionnaire study [15] evaluating the needs and requirements of designing mobile interventions for patients with PAD.

The TrackPAD pilot study was designed as a 2-armed randomized controlled trial and included patients with diagnosed and symptomatic PAD. It is a closed parallel-group trial (control and intervention groups were assessed simultaneously), with blinded assessors and face-to-face assessment components and a 3-month follow-up. Besides information regarding the pilot study, a call for participation was announced in a local newspaper (Westdeutsche Allgemeine Zeitung, local section for Essen and Duisburg) with the contact information provided, including the phone number and email address (trackPAD@uk-essen.de). In addition, potential participants were actively solicited during their outpatient clinic visits or their inpatient stay at the Department of Cardiology and Vascular Medicine, University Clinic of Essen. Willing patients were asked to register for the pilot trial at the front desk of the outpatient clinic.

#### Randomization

After screening based on inclusion and exclusion criteria and obtaining written informed consent, participants were randomized into 2 groups by the Center for Clinical Studies in Essen using the TENALEA software. The control group included participants with standard care and no further mobile intervention. The intervention group included participants receiving standard care and additional mHealth-based self-tracking of their physical activity using TrackPAD. The participants were stratified based on their 6-minute walking test (distances less than 362 meters, between 362 and 430 meters, and more than 430 meters) to ensure an even distribution of the walking speed between the two groups. After the randomization process, participants were not replaced, regardless of the reason for exclusion.

Both groups were strongly advised to continue with their SET according to the current standard guidelines [4]. Participants of the intervention group received additional access to the TrackPAD app, which complemented the patients’ current treatment. The TrackPAD app was freely accessible to the intervention group. Besides the support provided during the installation procedure, the app did not require further technical maintenance. The only external contact during the follow-up occurred if participants requested technical support. A nonphysician member of the study team helped participants.

The baseline and follow-up examinations took place at the Department of Cardiology and Vascular Medicine outpatient clinic. They included a 6-minute walking test and a measurement of the ankle-brachial index (ABI). The ABI Measurements were conducted using a Doppler probe on the tibial and anterior artery locations. According to the current European Society of Cardiology (ESC) guideline, the highest value was used for calculation and divided by the highest systolic brachial Doppler pressure [4].

The patients were asked to fill out a questionnaire package at both time points, including self-reported physical activity, demographic characteristics, and the PAD-QoL questionnaire. The PAD-QoL was translated into German by a native speaker and was pretested on 5 PAD patients not included in the study sample. The pretest did not reveal the need for any adjustments.

#### Inclusion Criteria

Main inclusion criteria were diagnosed and symptomatic PAD of the lower extremities, defined as Fontaine stage IIa or IIb. Fontaine stage IIa indicated intermittent claudication with a walking distance of more than 200 meters, whereas Fontaine stage IIb indicated intermittent claudication with a walking distance of fewer than 200 meters [4]. Additionally, patients must have a personal smartphone suitable for downloading and using the TrackPAD app (IOS version greater than 11.0 or Android version greater than 5.0). A detailed list of the inclusion and exclusion criteria is shown in Textbox 1.

**Inclusion and exclusion criteria. ABI: ankle-brachial index; PAD: peripheral arterial disease; NYHA: New York Heart Association; CCS: Canadian Cardiovascular Society.**

**Inclusion Criteria**

- 18 years of age or older
- Diagnosis of lower extremity PAD based on either an ABI greater or equal to 0.9 in at least one leg, invasive or noninvasive imaging of stenotic lower extremity artery disease, or endovascular or surgical revascularization of lower extremity artery disease
- PAD Fontaine stage 11a/b
- Smartphone with the capacity to use TrackPAD (Android version greater than 5.0 or IOS version greater than 11.0)
- Written informed consent prior to any study procedures, including a specified follow-up evaluation
- Best-medical treatment in the last 2 months per standard guidelines

**Exclusion Criteria**

- Wheelchair-bound, use of walking aid, or walking impairment due to another cause than PAD
- Below or above-knee amputation
- Acute or critical limb ischemia
- PAD Fontaine Stage I, III, or IV
- No German knowledge
- Severe cognitive dysfunction
- Congestive heart failure with NYHA III-IV symptoms
- Active congestive heart failure requiring the initiation or up-titration of diuretic therapy
- Angina pectoris with CCS class 3 to 4 symptoms, myocardial infarction, or stroke in the last 3 months
- Active arrhythmia requiring the initiation or up-titration of anti-arrhythmic therapy
- Severe valve disease

#### TrackPAD App

**Figure 1:**
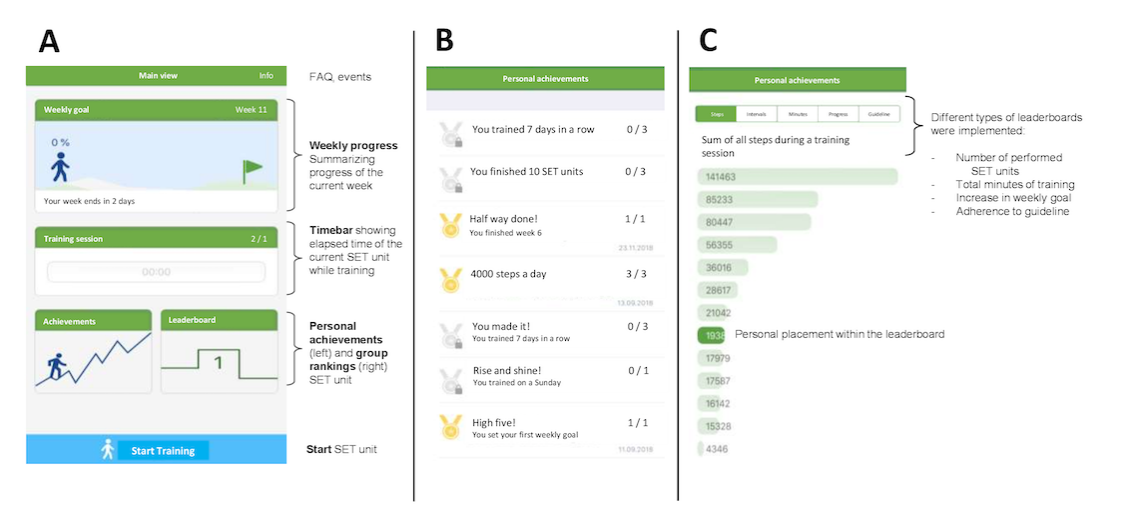
Main views of the TrackPAD-app.

The mobile intervention TrackPAD was designed by Rocket Apes GmbH. There were no associations between the authors and the developer. Moreover, TrackPAD was only designed for study purposes and not commercial use. We did not change any content during the study period, and all content was frozen during the trial. The only dynamic component was the leaderboard, which was adjusted based on the training sessions performed by the participants. The participants set their weekly goal of SET units at the beginning of each week. As recommended by the 2017 ESC guideline [4], each unit included 30 minutes of SET. If participants did not go through an entire unit at once, there were 3 different options: taking breaks, continuing the unit after recovery, or quitting prematurely. After completing each unit (fully completed 30 min or not), user feedback was requested (Figure 1; see Feedback after SET unit).

To account for a PAD-tailored solution, we included the following features (Figure 1):

1. Weekly goal adaptation: The app suggested a new weekly goal using an internal algorithm based on the completion rate of a user’s SET units during the previous week.
2. Feedback after SET units: The feedback after each SET unit contained PAD-specific information regarding leg pain levels, breathing, and overall exhaustion. Patients had to respond by choosing between 1 (minimum leg pain, no restriction in breathing, or minimum exhaustion) and 10 (maximum leg pain, maximum restriction in breathing, or maximum exhaustion) for each item.
3. Claudication reminder: Each SET unit started with a short reminder that the walking pace and incline must be adapted to reach a certain level of claudication to extend the PWD sustainably. The reminder popped up when each new SET unit was initiated and needed to be actively confirmed.
4. Personal achievements: The personal progress of each user was recorded to unlock achievement medals (eg, a notable increase in users’ physical activity, activity performed during public holidays, or successes like an increase of performed SET units per week).
5. Leaderboard: The leaderboard contained different categories (ie, number of steps in single training sessions, number of completed training sessions, total minutes of physical activity, and percent increase of physical activity). The different leaderboards showed individual placements compared to other users using TrackPAD.
6. Patient events: Information on upcoming Department of Cardiology and Vascular Medicine patient events focusing on vascular diseases were stored and easily accessible via the main menu.
7. PAD‑FAQ: An FAQ section was included to address common technical issues, important contact information, and general training advice. Instructions in case of increasing or new pain during the training were also included.

### Ethics Approval and Consent to Participate

The local ethics committee of the University of Duisburg-Essen (18‑8355‑BO) approved this study. Written informed consent was collected from each participant before any study procedures, and contact information was delivered to each participant. Any changes will be communicated to the ethics committee. The pilot study started at the beginning of November 2018 and ended in March 2019.

### Data Collection and Security

Data were stored on an encrypted European server. No personalized data were shared with the developer, and they were only accessible to the study team.

### Sample Size Considerations and Statistical Analysis

To allow for missing data and loss to follow up, we aimed to recruit 23 to 25 participants per study arm. The results achieved an estimated power of *t*=0.46 (post hoc power analysis; Cohen *d=*0.5; *P*=.05; *F*_1,46_=1.157). We used a two-tailed *t*-test, and the enrollment goal was 20 participants each for the intervention and control groups. *P*<.05 was estimated as the significance threshold. For sample size consideration and statistical analysis, we used R (version 3.6.0). To account for the heterogeneity of the walking distance to be covered, the analysis was performed separately for Fontaine stage IIa and IIb. The regression model was estimated by ordinary least squares and a differences-in-differences approach.

## Results

### Study Population and Baseline Characteristics

**Figure 2:**
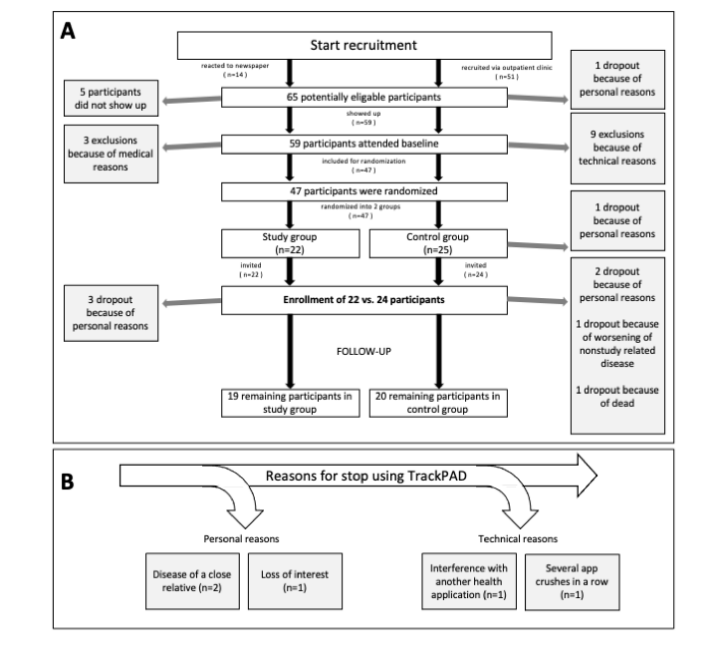
Quantitative development of screened patients including reasons for dropouts and exclusions.

After screening and randomization, we included 46 participants in the pilot study, of whom 22 (48%) were randomized to the intervention group, and 24 (52%) were randomized to the control. During the follow-up, 7 (15%) participants dropped out, mainly due to personal reasons. For example, 5 (11%) participants withdrew due to the severe illness of a close relative, and 2 (4%) participants dropped out as a result of either worsening of a nonstudy-related disease or death (Figure 2; see Panel A). Table 1 shows a summary of the remaining participants’ baseline characteristics.

**Table 1:** Patient characteristics at baseline.

	Intervention group (n=19)	Control group (n=20)	*P* value
Age (years), mean (SD)	64.6 (9.8)	65.6 (7.7)	.72
Sex (male), n (%)	12 (63)	9 (45)	.34
Obesity (BMI > 30 kg/m^2^), n (%)	5 (26)	1 (5)	.16
Prior MI^a^, n (%)	2 (11)	3 (15)	.85
Hypertension, n (%)	12 (63)	16 (80)	.41
Diabetes, n (%)	4 (21)	6 (30)	.21
Hyperlipidemia, n (%)	12 (63)	13 (65)	.42
Previous peripheral intervention, n (%)	8 (42)	5 (25)	.26
Previous peripheral bypass graft, n (%)	3 (16)	5 (25)	.68
Previous PCI^b^, n (%)	4 (21)	6 (30)	.69
Heart failure, n (%)	2 (11)	3 (15)	.85
Coronary arterial disease, n (%)	6 (32)	9 (45)	.51
Active/Former smoker, n (%)	6/11 (32/58)	8/10 (40/50)	.89
Fontaine stage IIa, n (%)	12 (63)	14 (70)	.44
Fontaine stage IIb, n (%)	7 (37)	6 (30)	.85
6-minutes walking distance (meters), mean (SD)	407 (80.8)	390.1 (66)	.35
ABI^c^	0.75 (0.21)	0.73 (0.18)	.46
Reported physical activity (days per week), mean (SD)	2.4 (1.4)	2.3 (1.9)	.35

^a^MI, myocardial injury.

^b^PCI, percutaneous coronary intervention.

^c^ABI, ankle-brachial index.

### Increase in the 6-minute Walking Distance as a Primary Outcome

Of the 20 participants who increased their 6-minute walking distance at follow-up, 18 (90%) belonged to the intervention group using TrackPAD. The remaining participant in the intervention group did not change his covered distance at follow-up. In contrast, except for 2 (10%) participants, 18 (90%) participants in the control group showed decreased 6-minute walking distance at follow-up.

The mean distance covered in the 6-minute walking test showed a significant increase in the intervention group overall (83.0 meters, SD 72.2), whereas the mean walking distance of the control group decreased on average (–38.8 meters, SD 53.7; *P*<.001).

Both Fontaine stages showed similar trends, but the mean distance increase for the less progressed Fontaine stage IIa was more pronounced (intervention group: 97.0 meters, SD 78.6 vs. the control group: –35.3 meters, SD 55.9; *P*<.001). The Fontaine stage IIb showed a slight increase in mean walking distance for the intervention group (59.0 meters, SD 57.0) compared to the control group (–7.0 meters, SD 52.2), but it was still significant (*P*=.01).

TrackPAD was linked to a mean increase in the 6-minute walking distance of the intervention group, regardless of the Fontaine stage (95% CI 48.2-117.8). In contrast, the control showed either a slight or missing increase (95% CI –63.9-3.6). In total, the difference between both means was 121.8 meters (Fontaine stage IIa: 132.3 meters; IIb: 106.4 meters). Depending on the Fontaine stage, this resulted in a 17% (IIb) to 23% (IIa) increase of the covered distance at follow-up (Table 2).

**Table 2:** Differences in the 6-minute walking distance within and between study and control group after 3 months of follow-up.

	Fontaine IIa (n=26)		Fontaine IIb (n=13)		Fontaine IIa, IIb (n=39)	
	Study (n=12)	Control (n=14)	Study (n=7)	Control (n=6)	Study (n=19)	Control (n=20)
Difference in mean^a^ (meters)	97.0	–35.3	59.4	–47.0	83.0	–38.8
Median (meters)	89.9	–22.0	30.0	–22.5	60	–22.0
SD (meters)	78.6	55.9	57.0	52.2	72.2	53.7
95% CI^b^ (meters)	47.0-147.0	–67.5-3.0	6.3-111.8	–101.8-7.8	48.2-117.8	–63.9-3.6
Difference in mean between both groups (meters)	132.3		106.4		121.8	
SD (meters)	135.5		71.6		176.4	
95%-CI^c^ (meters)	75.5-189.0		39.2-172.8		80.2-163.4	
*P* value	.01		.01		.01	

^a^ Positive mean indicates an improvement.

^b.^Difference between study and control group of the sub group.

^c^The true difference of the population between both groups.

A difference-in-difference regression with fixed effects for time (accounting for a progression of PAD) and individual participant (accounting for unobserved heterogeneity between the participants) estimating the percentage change in the treatment effect showed that the effect of receiving access to TrackPAD increased the 6-minutes walking distance about 28% (SE 0.04). This effect was significant to a confidence level of 99%.

### PAD-related Quality of Life

**Figure 3:**
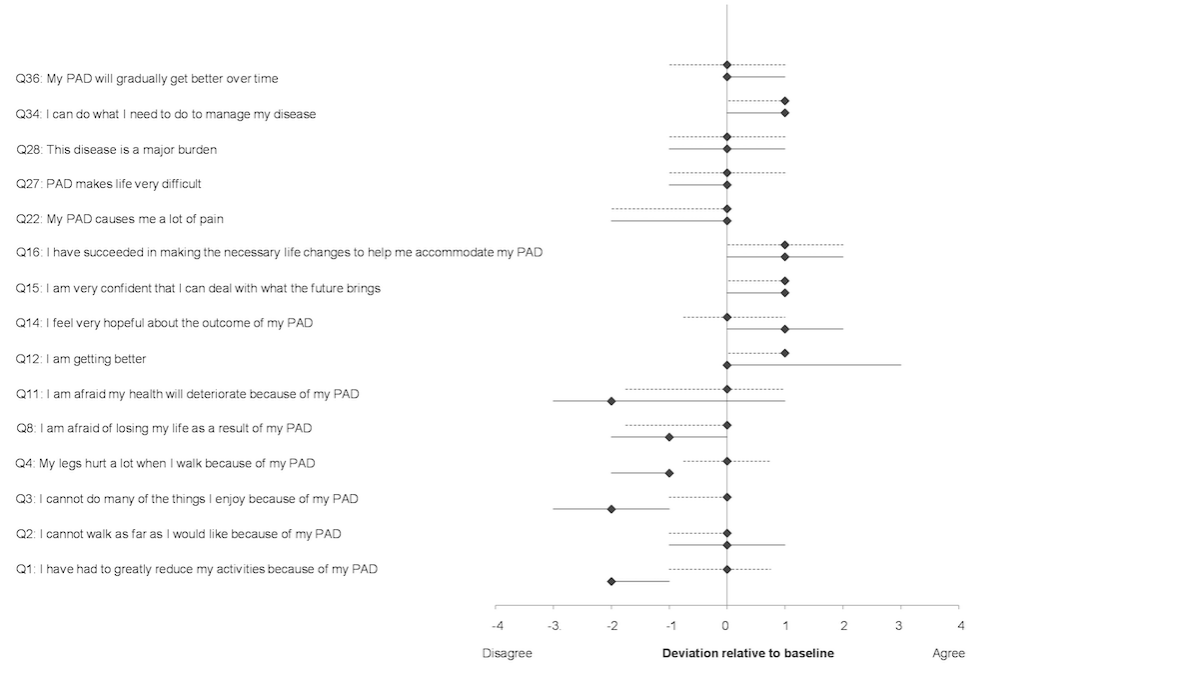
Excerpt of results from the PAD-QoL questionnaire survey [21]. Shown are the mean deviations relative to the baseline (diamond) and the 25th and 75th percentiles (lines). Study (solid line) and control group (dotted line) are plotted separately. PAD: peripheral arterial disease.

The PAD-related quality of life (PAD-QoL) was assessed by the PAD-QoL questionnaire at baseline and follow-up. No relevant differences were observed at baseline between both groups. However, at follow-up, significant changes were noted in 3 factors of the PAD-QOL, with the most extensive change evident in the “symptoms and limitations in physical functioning.” The intervention group reported reduced limitations in their daily activity: “I have had to greatly reduce my activities because of my PAD” (Q1, intervention group: –1.6 meters, SD 1.4 vs control group: –0.1 meters, SD 1.0; *P*=.01); “I cannot do many of the things I enjoy because of my PAD” (Q3, intervention group: –1.8 meters, SD 1.5 vs control group: –0.4 meters, SD 1.0; *P*=.01); and “My legs hurt a lot when I walk because of my PAD” (Q4, intervention group: –1.4 meters, SD 1.3 vs control group: 0 meters, SD 1.1; *P*=.01). The intervention group also showed a change in a single item of the factor “fear and uncertainty,” reporting a reduced fear of losing life because of PAD: “I am afraid of losing my life as a result of my PAD” (Q8, intervention group: –1.3 meters, SD 1.5 vs control group: –0.3 meters, SD 1.5; *P*=.048), and a change in the section “positive adaptation”: “I feel very hopeful about the outcome of my PAD” (Q14, intervention group: 1.2 meters, SD 1.1 vs control group: 0.2 meters, SD 1.4; *P*=.02; Figure 3).

Overall, changes in the PAD-QoL over the 3 months of follow-up showed a less intense subjective symptom perception and fewer limitations in daily life among the intervention group.

### Reported Physical Activity

To compare the two groups in terms of physical endurance at baseline, we recorded the reported physical activity. Both groups did not differ in days of physical activity per week (intervention group: 2.9 days per week, SD 2.8 vs control group: 2.4 days per week, SD 1.9; *P*=.44). Both groups had participants who were active for a median of 30 to 60 minutes (intervention group: n=9, 20% vs control group: n=4, 9%). In total, 12 participants (26%) were active for more than 60 minutes (intervention group: n=3, 7% vs control group: n=9, 20%). Among participants who exercised for less than 30 minutes weekly, 5 (11%) participants trained between 10 and 30 minutes weekly (intervention group: n=1, 2% vs control group: n=4, 9%), and 9 (20%) participants exercised less than 10 minutes weekly (intervention group: n=6, 13% vs control group: n=3, 7%).

At follow-up, 37 (80%) participants reported an increase in their weekly physical activity (intervention group: n=15, 33% vs control group: n=16, 35%), resulting in a comparable rise in physically active days per week in both groups (intervention group: plus 0.3 days per week, SD 3.5 vs control group: plus 0.4 days per week, SD 2.6; *P*=.93).

### App Evaluation

#### App Usage

We considered intervention participants as active users if they performed at least 1 weekly training. During week 1, every participant was active. A dip from 19 (100%) to 14 (74%) active users was observed in week 2, increasing to 17 (89%) active users in week 3. During the following weeks, the activity remained stable, with 14 to 15 (70% to 75%, respectively) active users from week 5 to 12 (Table 3). During the 12 weeks of follow-up, the number of training sessions per week stayed roughly the same for the participants that remained active users.

**Table 3:** TrackPAD-app usage of the intervention group during the 12 weeks of follow-up.

Week	1	2	3	4	5	6	7	8	9	10	11	12
Total training sessions (units)^a^	66	48	53	74	60	61	49	57	58	51	55	48
Active user^b^ (n)	19	14	17	16	15	15	15	14	15	14	14	15
Intervals per training session (units), mean^c^ (SD)	2.5 (2.8)	2.8 (2.2)	2.6 (1.7)	2.0 (1.2)	1.8 (1.2)	2.3 (1.6)	2.7 (2.8)	2.4 (2.0)	2.2 (2.3)	2.1 (1.9)	2.5 (2.1)	2.7 (4.1)

^a^Total number of recorded training sessions for the respective week as assessed by the TrackPAD-App.

^b^Active user with at least one training interval in the corresponding week.

^c^Mean number of intervals during one training session. Each training session could be paused if necessary, resulting in each training session being subdivided into several intervals.

The reasons for discontinued TrackPAD use by the nonactive users (n=5, 26%) from week 5 onward were assessed at follow-up. Reasons for discontinued TrackPAD use were related to personal circumstances (n=3, 16%) and technical issues (n=2, 11%). Of the 2 participants who stopped using TrackPAD for personal reasons, 1 was due to the illness of a close relative, and the other lost interest. One participant stopped using TrackPAD due to reported interference between the TrackPAD app and their Samsung Health app, and another participant stopped the training sessions due to several sequential app crashes (Figure 2; see Panel B).

#### User Feedback

**Figure 4:**
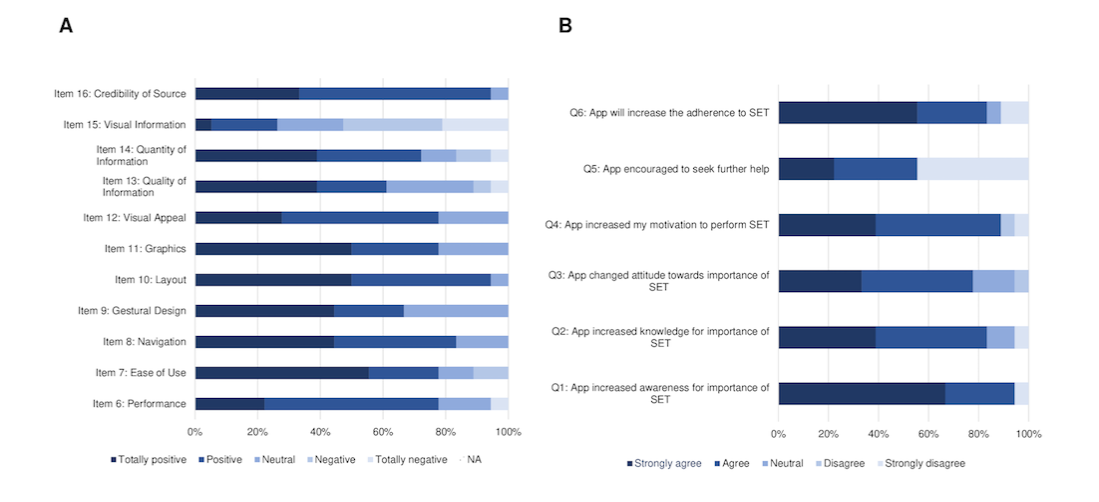
Participants' statements regarding the trackPAD app in terms of functionality, aesthetics and information according to the user version of the Mobile Application Rating Scale [22].

The vast number of questions regarding functionality, aesthetics, and informational content of TrackPAD were reported as positive to extremely positive (4 or 5 stars out of 5; Figure 4; see Panel A). However, the visual information provided within the app showed potential for improvement (Figure 4; see Panel A, Item 15); for example, the plausibility and correctness of descriptions represented by pictograms or pictures. Participants described this item mainly as “largely unclear.” Only 5 (25%) participants described the visual information as “mostly clear”(n=4) or “absolutely clear” (n=1).

The users’ feedback also included questions regarding the perceived impact of the TrackPAD with respect to their PAD disease (Figure 4; see Panel B). Only 1 (6%) user disagreed, stating the app had not changed their awareness of SET (Q1). The other participants reported that the app had significantly increased their motivation to perform SET (Q4) and their compliance to SET (Q6). They also stated that using the app changed their attitude regarding SET (Q3) and increased their knowledge about SET (Q2).

**Figure 5:**
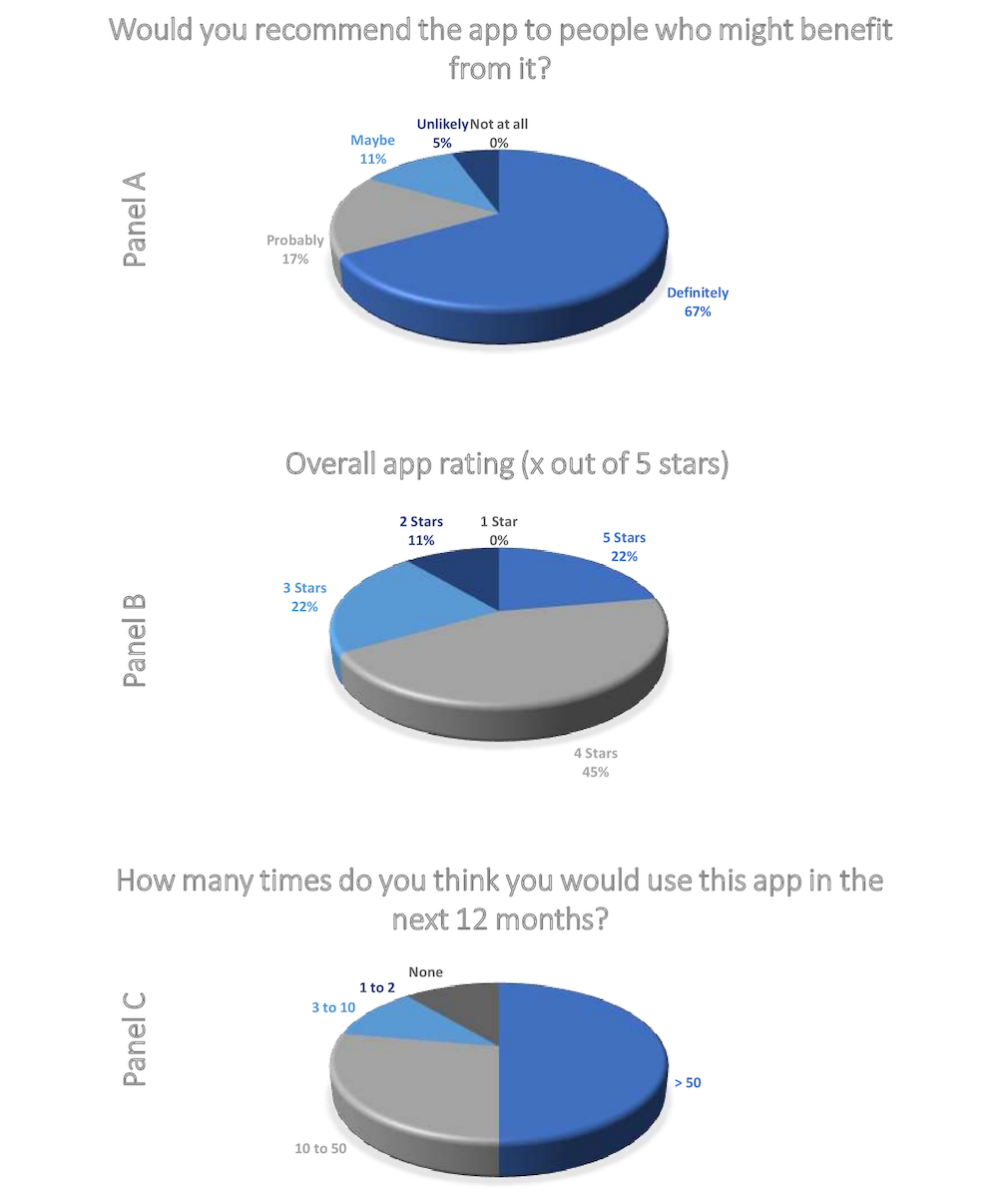
App rating of the study group after study end according to the user version of the Mobile Application Rating Scale [22].

Most users evaluated the app in all of the 3 categories positively. Only 3 (17%) users would “maybe” or are “unlikely” to recommend the app to people with existing PAD disease. Supporting the positive evaluation illustrate in Panel A (Figure 5), 13 (68%) users rated the app with at least 4 out of 5 stars (Figure 5; see Panel B). Future app use, at least every week, was reported by 10 (53%) users.

The data underlying this article will be shared at reasonable request to the corresponding author.

## Discussion

The implementation of novel technologies, specifically mobile interventions, can substantially change the landscape for the treatment of CVD [12,30]. General benefits of mHealth technologies include the wide reachability and the possibility of continuous access [31]. Although PAD represents a subgroup of CVD, patient characteristics and disease-specific requirements differ substantially from those patients with other CVD. Therefore, disease- and patient-tailored solutions are essential to the development of mobile interventions. One significant difference between the PAD population and patients with other CVD is the older age and the fact that the patient-centered development process needs to be expanded by one additional dimension. Previous studies already explored the use and acceptability of mobile technologies in health care related to the users’ age and identified age as an important factor in the design of mobile interventions, requiring greater technical support and reporting lower acceptability of using mobile technologies [32,33]. As such, there are measurable influences on intermediate outcomes (eg, increased satisfaction with care) and health outcomes (eg, better metabolic control) [15,34-36].

SET is one of the most relevant interventions in the conservative treatment of PAD, but barriers to exercise are still high. Besides low motivational aspects, intermittent claudication limits the sustainability of regular SET performance. Moreover, the requirements of primary care for patients with PAD focus on other priorities other than CVD in general [15,30]. To meet this specific patient population's needs and requirements, we proposed using a PAD-tailored mobile intervention to encourage the SET performance in patients with PAD.

Mobile technologies are increasingly used for health purposes, even among older adults who have demonstrated a lower uptake of technologies compared to younger people [37]. Although these technologies have the potential to assist in care coordination activities, like regular SET performance, most mobile apps are not designed specifically for this population which has complex health care needs and is older than the typical app user. The activity recognition mechanism of most mobile apps cannot accommodate the wide range of human movement linked to mobility impairment.

In this study, we gathered TrackPAD use input from the patients’ perspective, and we observed a high level of user acceptance. Overall, we found satisfaction in terms of functionality, aesthetics, and informational content. Studies combining eHealth and PAD are rare, but the same trend of mobile technology user acceptance was observed in patients with noncommunicable diseases. A review of eHealth interventions for cancer survivors showed mobile interventions are promising tools [38]. Future work will need to examine the extent to which personalized activity recognition can support the diversity of movement.

Improvement was demonstrated through the visual information within TrackPAD and the clear assignment of pictograms or pictures. The weakness of the gestural concept resulted from the advanced age of the user group, which is often inexperienced in using mHealth and requires an age-adapted presentation [39]. Besides relevant barriers for older adults, lack of desire, costs, privacy and security considerations, visual acuity, and hand-eye coordination were important factors with respect to the acceptance of telehealth interventions [40]. These barriers will be adapted accordingly to improve the TrackPAD app following a patient-centered approach.

Since we designed a platform for both iOS and Android, some technical issues occurred due to the different technical implementations of the provider. The various mechanisms for counting steps presented a considerable challenge in designing a comparable app for both platforms. Depending on the manufacturer, step counts work either over a physical hardware mechanism and a software-based solution. This issue might become less relevant when it comes to personal use [41,42], but it also limits the analysis within a clinical trial. Because of the low number of smartphones that use the software-based solution, this issue did not occur in earlier tests. However, the disproportionate share of older mobile phones lacking a physical hardware mechanism within the intervention group revived the issue. In further trials, the inclusion of newer operating versions of Android mobile phones should be considered since this issue was only found in Android-based operating systems.

The disadvantages of simple activity tracking are known and common limitations in studies. The performance of systems trained with data in the laboratory setting substantially deteriorates when tested in real-life conditions [43]. Possible solutions might be user-calibration processes or the use of specified study-related devices to gain comparability.

Comparing the 6-minute walking distance between both groups in our study, we saw a significant increase in the mean walking distance of 80 meters in the intervention group using TrackPAD. Remarkably, we did not find any decrease in the walking distance within the intervention group, whereas 90% (n=18) of the control group did worse at follow-up compared to baseline. One reason for the longer walking distance might be because of the younger age of the study participants. Previous studies reported a mean age of more than 70 years, whereas the intervention group using TrackPAD had a mean age of 64. The higher increase may also be due to comparatively minor restrictions since two-thirds of the participants were classified as Fontaine stage IIa (PWD of more than 200 meters). The ease of initiating exercise among the Fontaine stage IIa patients with PDA compared to patients with higher Fontaine stages might be linked to better endurance during exercise and higher motivation in general. Moreover, the small sample size allows for substantial individual changes within the intervention group, leading to an upward deviation.

Although the covered distance in the 6-minute walk test only increased significantly in the intervention group, the self-reported physical activity increased in both groups at follow-up. An accurate assessment of physical activity using the PDA-QoL questionnaire seems questionable in the entire study population and has previously been described as a common issue [44,45]. Digital interventions also increase the potential to track background activity (ie, receiving objective statements in terms of physical activity) and will be considered in future trials. The recording of activity highs and lows throughout the day might also help identify optimal time points to send digital motivation notifications. It is important to note that messages can also decrease productivity if delivered at the wrong time points. Algorithms based on collected personalized information in smartphones might reduce the number of poorly-timed interruptions [46].

We also observed an increase in PAD-QoL regarding “symptoms and limitations in physical functioning” within the intervention group. The association between increased physical activity and an increased PAD-QoL has been reported in other studies [47-49]. In addition, the increased 6-minute walking distance was linked to better physical aspects of quality of life in participants with intermittent claudication, supporting its value as an outcome measure.

The main limitation of this study was the small sample size of the intervention group. Since we have analyzed some patient characteristics (ie, Fontaine stage IIa and IIb) separately, the sample size per group decreased even further. However, the Fontaine stage allowed us to control for the differences in the participants’ physical capability. Although we saw a relevant change in the primary outcome variable after follow-up, recordings of background activity during the follow-up period were available due to privacy restrictions. Based on the study design of this pilot, no blinding of the study participants was feasible, and motivational differences must be considered. Further research is needed to address this issue.

Using the smartphone–based tool TrackPAD, we found a significant increase in the mean 6-minute walking distance at follow-up, indicating a prognostically relevant change in walking ability in patients with moderate PAD. TrackPAD also bolstered a shift in the subjective symptom perception and fewer noticed limitations in terms of PAD-QoL. Thus, the TrackPAD app seems feasible and suitable for the target group of patients with PAD in terms of SET performance. Participants substantially valued the experience of using an app in the management of their care. Still, a further adaption of the visual presentation and the gestural concept that follows a patient-centered approach is needed.

## Appendix

Multimedia Appendix 1 CONSORT-eHEALTH checklist (V 1.6.1).

## Abbreviations

ABI: ankle-brachial index
CAD: coronary artery disease
ESC: European Society of Cardiology
mHealth: mobile health
PAD: peripheral arterial disease
PAD-QoL: peripheral arterial disease–related quality of life CVD: cardiovascular disease
PWD: pain-free walking distance
QoL: quality of life
SET: supervised exercise training
